# Change in exposure of children to second-hand smoke with impact on children’s health and change in parental smoking habits after smoking ban in Bavaria – a multiple cross-sectional study

**DOI:** 10.1186/s12889-021-12130-8

**Published:** 2021-11-20

**Authors:** Mohammed El Sharkawy, Stefanie Heinze, Lana Hendrowarsito, Alisa Weinberger, Jonas Huß, Uta Nennstiel, Caroline Herr, Susanne Kutzora, for the GME Study Group, Wiltrud Doerk, Angelika Pfister, Ro S E Earie Sit-tig, Winfried Strauch, Heidi Thamm, Anita Wunder, Tatjana FrießHesse, Franziska Lang, Dagmar Rudolph, Roland Schmid, Gudrun Winter, Isabella Bockmann, Christine Gampenrieder, Margot Motzet, Elisabeth Schneider, Traudl Tontsch, Gerlinde Woelk, Sylvia Kranebitter, Heidi Mayrhofer, Gertraud Rohrhirsch, Brigitte Weise, Luisa Wolf, Kornelia Baranek, Gitte Koch-Singer, Maximilian Kühnel, Ladan Baghi, Otmar Bayer, Rüdiger von Kries, Gabriele Bolte, Hermann Fromme, Annette Heißenhuber, Lana Hendrowarsito, Caroline Herr, Martina Kohlhuber, Joseph Kuhn, Bernhard Liebl, Anja Lüders, Nicole Meyer, Christine Mitschek, Gabriele Morlock, Michael Mosetter, Uta Nennstiel-Ratzel, Dorothee Twardella, Manfred Wildner, Angelika Zirngibl

**Affiliations:** 1 Institute for Medical Information Processing, Biometry and Epidemiology – IBE, LMU Munich, Munich, Germany; 2 Pettenkofer School of Public Health, Munich, Germany; 3 Department for Occupational and Environmental medicine, Bavarian Health and Food Safety Authority, Pfarrstraße 3, Munich, Germany; 4 Institute and Outpatient Clinic for Occupational, Social and Environmental Medicine, Hospital of the University of Munich, Ziemssenstr. 1, 80336 Munich, Germany; 5 Bavarian Health and Food Safety Authority (LGL), Veterinärstraße 2, 85764 Oberschleißheim, Germany

**Keywords:** Active smoking, Children’s health in Bavaria, Second-hand smoking, Smoking ban in Bavaria, Respiratory diseases in children, Maternal smoking during pregnancy

## Abstract

**Background:**

Concerns about smoking displacement from public places to private amenities aroused following smoking ban implementation in Bavaria in 2008. We analysed children’s exposure to second-hand smoke (SHS) before and after the ban, its effect on children’s health and prevalence of active smoking in adults.

**Methods:**

Six cross-sectional surveys (*n* = 32,443) on pre-school children in Bavaria were analysed, two surveys before the smoking ban in years 2004 and 2005 (S1 and S2) and four after the ban in 2008, 2012, 2014 and 2016 (S4, S6, S7 and S8). Using multivariable logistic regression, we analysed change in children’s intra- and extrauterine SHS exposure and its adverse health effects (Asthma, wheezing, bronchitis and neurodermatitis) as well as change in parental active smoking.

**Results:**

The response rates were 78% for S1, 73% for S2, 61% for S4, 62% for S6, 56% for S7 and 54% for S8. Odds of parents never smoked at home in presence of children increased significantly from before to after the ban with odds ratios (OR) 1.17 (CI_95%_ 1.01–1.35), 1.65 (CI_95%_ 1.39–1.95), 2.85 (CI_95%_ 2.32–3.51), 2.24 (CI_95%_ 1.84–2.72) and 3.66 (CI_95%_ 2.89–4.63) for S2, S4, S6, S7 and S8, respectively with S1 as reference. Compared to S4, odds of parents who were not actively smoking is significantly higher in S7 (OR = 1.13 (CI_95%_ 1.03–1.24)) and S8 (OR = 1.24 (CI_95%_ 1.13–1.36)). The odds of mothers who never smoked during pregnancy increased over time with OR = 1.22 (CI_95%_ 1.06–1.40) for S2 and 1.57 (CI_95%_ 1.33–1.86) for S8 compared to S1. Adverse health effects related to children’s exposure to SHS are significantly less in S8 compared to S1.

**Conclusion:**

After 11 years of smoking ban in Bavaria, smoking displacement to homes was disproved. Exposure of children to SHS intrauterine and at home is decreasing. Number of parents who are not actively smoking is increasing over time. Prevalence of health problems in children related to exposure to SHS is decreasing.

**Supplementary Information:**

The online version contains supplementary material available at 10.1186/s12889-021-12130-8.

## Background

Adverse health effects from exposure to second-hand smoke (SHS) has been first described since 1928 [1]. Exposure of non-smoking adults and children to SHS increases health burden. According to a study performed in 2004 [2], 40% of children, 33% of male non-smokers, and 35% of female non-smokers were exposed to SHS worldwide. Estimated 603,000 deaths was attributable to SHS exposure in 2004, constituting about 1.0% of worldwide mortality, 28% of which are children [3]. Exposure of pregnant women to SHS – especially during the third trimester [4] - was reported to have multiple adverse foetal outcomes as stillbirth [5], low birth weight [6, 7], preterm delivery [7], asthma and allergy related symptoms. Also maternal smoking during pregnancy as a source of intrauterine child SHS exposure, proved to cause infancy and childhood adverse health effects as low birth weight [8], childhood asthma [9], Obesity [10] in addition to persistent epigenetic changes [11]. Additionally, exposure of new-born and young children to SHS was associated with impaired neurodevelopment and behaviour [12–14], wheezing [15, 16], asthma, bronchitis and nocturnal cough [17, 18]. In this context, considering socially deprived groups, single parents and parents with lower socioeconomic status, are more likely to expose their children to SHS than other more privileged social groups [19, 20].

The health hazards attributed to SHS exposure strongly urge the efforts for smoking cessation in active smokers as well as ensuring smoke-free environment for non-smokers. Following the WHO Framework Convention on Tobacco Control (WHO FCTC) in 2007, far-reaching laws to protect non-smokers were imposed in Germany’s 16 federal states [21]. The federal state of Bavaria has implemented total smoking ban in all closed restaurants, bars and cafés in 2008 and further imposing a total ban in all indoor private and public places in 2010 [22].

Although such a ban is essential to guarantee a smoke-free environment in public places for non-smoking adults and children, concerns were raised about smoking displacement to enclosed private places where non-smokers live. Nevertheless, most studies disproved the displacement hypothesis after smoking ban implementation in many countries [23–26], with some studies showing parental smoking at home decreased significantly with more self-implemented smoking restrictions at home especially in presence of children [26]. This decrease in smoking could be directly linked to the smoke-free legislation in some studies [27] or observed as additional voluntarily-introduced home bans in others [28].

Moreover, evidence shows a decreasing trend in active smokers over years following smoking bans. A Cochrane review published in 2010 investigating the effect of implementing institutional smoking bans, found evidence on effect of settings-based smoking policies on reducing smoking rates as well as reduced mortality rates and reduced exposure to SHS [29]. Another Cochrane review published in 2016 found that legislative smoking bans clearly lead to a reduction in exposure to SHS, strong evidence from the included studies supports a decline in admissions for acute coronary syndrome and a weak trend for a decrease in active smoking [30]. These results come in line with the intended outcomes for the smoking ban with some other studies showing support for extending the ban to include more amenities especially in presence of children [31, 32].

Clear evidence on the health benefits of this decrease in exposure to SHS is increasingly emerging particularly for children, showing reductions in low birthweight deliveries, preterm birth and asthmatic exacerbations in Bavaria [33] as well as internationally [34]. A systematic review in 2014 by Been et al. including 2.5 million children found that smoke-free legislation is associated with substantial reductions in preterm births and hospital attendance for asthma, together with the health benefits in adults [34].

In this multiple cross-sectional study, we investigate the change in smoking habits of parents of children in Bavaria qualifying for first school entry year using data from surveys performed before and after the smoking ban imposed in year 2008, we investigate the odds of parental smoking in presence of children, maternal smoking during pregnancy and parental active smoking. We also investigate the association of this change on prevalence of asthma, bronchitis, neurodermatitis and wheezing as adverse health effects for children’s exposure to SHS.

## Methods

### Data collection and study design

The GME surveys are cross-sectional survey that have been conducted every 2 years since 2004 in Bavaria, whose inhabitants ranged from 12,5–13 million during the time span of the GME surveys, conducted since 2004 till 2016. With the help of questionnaires, which are distributed to parents of preschool children during the school entrance examinations, standardised data on the health and living environment of the children are collected. In contrast to the compulsory school entrance examinations, participation in the GME is voluntary. In carrying out the GME, the Bavarian State Office for Health and Food Safety (LGL) cooperates with external partners from the university sector and the local health authorities. The Bavarian Authority for Health and Food Safety regularly sends questionnaires to health authorities of three urban cities (Munich, Ingolstadt, Bamberg) and three rural areas (Schwandorf, Günzburg, Bamberg rural) within the framework of the health monitoring units [35] in Bavaria, Germany. Parents of children aged 5–6 years were asked to voluntarily participate in filling the questionnaires along with their children’s compulsory qualifying school entrance examination. Six cross-sectional surveys were analysed with a total of 32,443 participants. The six surveys have 13 years’ time span, two surveys were performed before the smoking ban: S1 (2004) and S2 (2005), and four after the smoking ban: S4 (2008), S6 (2012), S7(2014), S8 (2016). Response rates were 78% for S1, 73% for S2, 61% for S4, 62% for S6, 56% for S7 and 54% for S8. More details about GME and data collection are described elsewhere [35]. The questionnaires were based on standard scales used in previous studies [36].

### SHS exposure and active smoking

Data from the six surveys was analysed for parental smoking at home in presence of children using a binary (yes, no) coded variable constructed from answer to the questions asking about parental smoking habits at home in presence of their children, with ‘No’ if none of the parents ever smoke at home in presence of children and ‘Yes’ if at least one of the parents smokes in presence of children. Parental active smoking was investigated using data from the four surveys after the ban (S4, S6, S7 and S8) using a binary coded variable (none of the parents currently smoke, at least one of the parents currently smokes) constructed from answers to questions asking about the smoking status of both parents. The two surveys before the smoking ban (S1 and S2) did not include questions asking about smoking status of parents. As an intrauterine source of child SHS exposure, maternal smoking during pregnancy was investigated by analysing data from two surveys before (S1 and S2) and two surveys after (S7 and S8) the ban, asking smoking mothers if they ever smoked during pregnancy using a binary (yes, no) variable.

### Adverse health effects in children related to exposure to SHS

We analysed four surveys (S1, S6, S7 and S8) with questions asking the parents if the child has ever been diagnosed with asthma, bronchitis or neurodermatitis by a doctor and about the number of wheezing episodes the child had in the last 12 months. Variables for being diagnosed by a doctor with asthma, bronchitis and neurodermatitis are dichotomized as ‘Yes’ if the question was answered with ‘yes, once’ or ‘yes, several times’ and ‘No’ if answered with ‘No, never’. For wheezing, a binary (no or less than four episodes in the last 12 months, four or more episodes in the last 12 months) variable was constructed, from responses ‘No, never’, ‘Yes, 1 to 3 episodes’, ‘Yes, 4 to 12 episodes’ and ‘Yes, more than 12 episodes’.

### Socio-demographics

Questions describing socio-demographic status across questionnaires were used to define variables as gender, urbanization (rural, urban), parental education (high, middle and low), employment status for mothers and fathers (> 15 h /week or full-time, < 15 h /week, inactively employed and unemployed), maternal partnership status (single, not single) and living in crowding conditions (yes, no) with crowding defined as more than one person per room or having < 20 m^2^ living space per person. Parental education is ‘high’ when one or both parents are holding a university entrance certificate, ‘middle’ when highest degree is high secondary school certificate and ‘low’ if holding low/no secondary school certificate. The “urbanisation” status for 1070 questionnaires in S7 was missing and was imputed by random assignment to one of the values (see supplementary, Methods section).

### Statistical analysis

Multivariable logistic regression was used to analyse change in active smoking, child domestic SHS exposure, maternal smoking during pregnancy as well for each adverse health effect as the outcome variables. A categorical time trend variable reflecting time change between questionnaires was used as a predictive variable in the regression model. This time trend variable was used as the exposure variable to detect the change in all measured outcome variables in two time-points before the ban (S1 and S2) and four time-points after the ban (S4, S5, S7 and S8), the point estimates of this time trend variable were extracted and described. Child’s gender, urbanization, parental education, employment of the mother, employment of the father, child’s birth-place and crowding were the variables used to adjust for change in outcome variable with change in the time trend exposure variable. S8 did not have information about mothers being single or not so the variable was not included in the regression analysis. To control for multicollinearity, a bivariate correlation analysis (Cramer’s V test) was performed to identify confounders with significant associations. None of the included variables were strongly correlated (taking Cramer’s V value > 0.30) so none of them was excluded from the regression model. Model selection was done using LASSO method for model features selection, a method that imposes a constraint on the model parameters that causes regression coefficients for some variables to shrink toward zero then removing all parameters. Variables with a regression coefficient equal to zero after the shrinkage process are excluded from the model. Child’s exposure to domestic SHS stratified by variables ‘single mother’ and ‘parental education’ is presented as bar charts (see supplementary). Group differences were checked for significance using the Rao-Scott chi-square test for complex samples. Differences are regarded statistically significant if the confidence intervals do not overlap or if the probability of error (p) takes on a value smaller than 0.05. All analytical procedures were performed using SAS 9.2 (SAS Institute Inc., Cary, NC, USA).

## Results

### Socio-demographics

A total of 32,443 parents were surveyed. Table 1 shows the socio-demographic characteristics for the six surveys conducted. In our data, rates for living in urban areas, parental education level and children being born outside Germany increased over time from S1 to S8, whilst living in crowding conditions and children living with single mothers did not change across the surveys.

**Table 1:** Socio-demographic characteristics of GME^a^ surveys

Survey (year of survey)	S1 (2004)	S2 (2005)	S4 (2008)	S6 (2012)	S7 (2014)	S8 (2016)
Total (N)	6350	6206	5336	5052	4732	4767
Characteristic	n (%)	n (%)	n (%)	n (%)	n (%)	n (%)
**Gender**						
Male	3319 (52.3)	3216 (51.8)	2738 (51.3)	2668 (53.0)	2418 (51.5)	2420 (51.3)
Female	3030 (47.7)	2989 (48.2)	2598 (48.7)	2370 (46.9)	2286 (48.3)	2296 (48.1)
Missing	1	1	0	14	34	51
**Urbanisation**						
Urbanised area	2640 (41.6)	2763 (44.5)	2598 (48.7)	2725 (54.0)	2512 (53.1)	2453 (51.5)
Rural area	3710 (58.4)	3443 (55.4)	2738 (51.3)	2327 (46.0)	2220 (46.9)	2314 (48.5)
Missing	0	0	0	0	0	0
**Parental Education**						
High	2322 (37.8)	2317 (38.6)	2127 (41.2)	2235 (46.7)	2295 (50.2)	2416 (52.7)
Middle	2105 (34.2)	2052 (34.2)	1812 (35.1)	1624 (33.9)	1425 (31.2)	1344 (29.3)
Low	1720 (28.0)	1630 (27.2)	1221 (23.7)	928 (19.4)	850 (18.6)	821 (17.9)
Missing	203	207	176	265	162	186
**Employment mother**						
Full-time or > 15 h/week	2123 (35.7)	2102 (35.8)	2177 (43.1)	2341 (49.8)	2309 (51.9)	2480 (55.1)
< 15 h/week	1373 (23.1)	1350 (23.1)	1081 (21.4)	925 (19.7)	847 (19.0)	779 (17.3)
Inactively employed	1994 (33.5)	1912 (32.6)	1539 (30.5)	1193 (25.4)	1073 (24.1)	1078 (23.9)
Unemployed	457 (7.7)	502 (8.6)	257 (5.1)	239 (5.1)	224 (5.0)	164 (3.6)
Missing	403	340	282	354	279	266
**Employment father**						
Full-time or > 15 h/week	5405 (93.3)	5365 (93.7)	4649 (94.7)	4389 (94.9)	4149 (95.2)	4193 (95.7)
< 15 h/week	62 (1.1)	53 (0.9)	48 (1.0)	52 (1.1)	35 (0.8)	41 (1.0)
Inactively employed	85 (1.5)	69 (1.2)	69 (1.4)	61 (1.3)	64 (1.5)	75 (1.7)
Unemployed	242 (4.2)	241 (4.2)	144 (2.9)	125 (2.7)	111 (2.6)	75 (1.7)
Missing	556	478	426	425	373	383
**Single mother**						
Yes	559 (9.1)	586 (9.6)	464 (8.9)	447 (9.2)	430 (9.3)	
No	5791 (88.3)	5515 (88.8)	4726 (88.5)	4413 (87.3)	4192 (88.5)	
Missing	180	105	146	192	110	
**Birthplace of child**						
Outside Germany	195 (3.1)	190 (3.1)	124 (2.3)	170 (3.4)	258 (5.5)	335 (7.1)
Inside Germany	6148 (96.8)	6001 (96.6)	5164 (96.7)	4818 (95.3)	4440 (93.8)	4380 (91.8)
Missing	7	15	48	64	34	52
**Crowding**						
Yes	1180 (19.2)	1160 (19.2)	920 (18.1)	948 (20.2)	879 (19.7)	881 (19.8)
No	4957 (78.0)	4929 (79.4)	4169 (78.1)	3738 (73.9)	3591 (75.8)	3564 (74.7)
Missing	213	177	247	366	262	322

^a^GME: For health monitoring units (Gesundheits-Monitoring-Einheiten)

### SHS exposure and actively smoking parents

Percentage of parents who reported they never smoke at home in presence of their children has increased with time across surveys from 90.7% in S1 to 97.5% in S8 (Table 2). Percentage of mothers who never smoked during pregnancy has also increased from 88.7% in S1 to 93.9% in S8. In surveys done after the smoking ban, number of parents reporting none of them is actively smoking has increased from 58.6% in S4 to 65.6% in S8. Stratified by being a single mother or not, children of single mothers are exposed more to domestic SHS from their mothers compared to children of non-single mothers. Percentage of mothers not smoking in the presence of children at home has significantly increased across surveys for both single and non-single mothers (*P* < 0.001) (See supplementary Fig. 1). Stratified by parental education level, parents with low education not exposing their children to domestic SHS are less compared to parents with medium or high education, although the increasing trend across surveys of not smoking at home in presence of children for parents with low education level is greater than in parents with higher education levels. The increase in not exposing children to domestic SHS across surveys is significant for all educational categories (*P* < 0.001) (See supplementary, Fig. 2).

**Table 2:** Prevalence of exposure of children to SHS* and active smoking parents

Survey (year of survey)	S1 (2004)	S2 (2005)	S4 (2008)	S6 (2012)	S7 (2014)	S8 (2016)
Total (N)	6350	6206	5336	5052	4732	4767
Characteristic	n (%)	n (%)	n (%)	n (%)	n (%)	n (%)
**Do you smoke in presence of children at home?**						
Never	5613 (90.7)	5584 (91.6)	4839 (94.2)	4593 (96.8)	4417 (95.9)	4529 (97.5)
Yes	575 (9.3)	512 (8.4)	297 (5.8)	153 (3.2)	188 (4.1)	116 (2.5)
Missing	162	110	200	306	127	122
**Did the mother smoke during pregnancy?**						
Never	5428 (88.7)	5431 (90.8)			4224 (91.6)	4381 (93.9)
Yes	692 (11.3)	551 (9.2)			385 (8.4)	285 (6.1)
Missing	230	224			123	101
**Does any of the parents smoke?**						
None of the parents smoke			3080 (58.6)	2978 (60.5)	2866 (62.7)	3025 (65.6)
At least one parent smoke			2172 (41.4)	1942 (39.5)	1703 (37.3)	528 (34.4)
Missing			84	132	163	155

*SHS: Second-hand smoking

### Adverse health effects in children related to exposure to SHS

The proportion of parents reporting their child was never diagnosed by a doctor with asthma, bronchitis or neurodermatitis has increased from 97.5% in S1 to 98.6% in S8 for asthma, 67.4% in S1 to 74.1% in S8 for bronchitis and 87.6% in S1 to 90.4% in S8 for neurodermatitis. Proportion of parents reporting their child had four or less wheezing episodes in the previous year has slightly increased from S1 to S8 (Table 3).

**Table 3:** Prevalence of adverse health effects in children related to SHS* exposure

Survey (year of survey)	S1 (2004)	S6 (2012)	S7 (2014)	S8 (2016)
Total	6350	5052	4732	4767
Characteristic	N (%)	N (%)	N (%)	N (%)
**Child ever diagnosed with asthma by a doctor?**				
Never	5114 (97.5)	4478 (97.2)	4111 (97.0)	4223 (98.6)
Yes	134 (2.6)	130 (2.8)	129 (3.0)	59 (1.38)
Missing	1102	444	492	485
**Child ever diagnosed with neurodermatitis by a doctor?**				
Never	5348 (87.6)	4381 (88.9)	3684 (88.1)	3885 (90.4)
Yes	760 (12.4)	549 (11.1)	499 (11.9)	411 (9.6)
Missing	242	122	549	471
**Child ever diagnosed with bronchitis by a doctor?**				
Never	3802 (67.4)	3215 (68.8)	3070 (72.1)	3218 (74.1)
Yes	1843 (32.7)	1459 (31.2)	1190 (27.9)	1125 (25.9)
Missing	705	378	472	424
**Child had wheezing episodes in last 12 months?**				
< 4 episodes	6085 (98.4)	4850 (98.5)	4495 (98.7)	4555 (99.2)
> = 4 episodes	97 (1.6)	72 (1.5)	61 (1.3)	35 (0.76)
Missing	168	130	176	177

### Regression analysis

For SHS exposure, the odds of children not exposed to domestic SHS from parents have significantly increased over time across surveys compared to reference survey S1. The odds of mothers who never smoked during pregnancy have increased significantly over time across surveys with OR = 1.22 (CI_95%_ 1.06–1.40) for S2 and 1.57 (CI_95%_ 1.33–1.86) for S8 compared to S1 although not significant for S7 compared to S1 (Table 4). For active smoking, parents reporting none of them is actively smoking also showed a significant increase across surveys with OR = 1.13 (CI_95%_ 1.03–1.24) for S7 and 1.24 (CI_95%_ 1.13–1.36) for S8 compared to reference survey S4 although not significant for S6 (Table 4). This indicates a decrease in children’s exposure to SHS from parents and decreasing prevalence of active smoking.

**Table 4:** Change in odds ratios (OR) for exposures and outcomes in children related to SHS* exposure across surveys

Survey (year of survey)	S1 (2004)	S2 (2005)	S4 (2008)	S6 (2012)	S7 (2014)	S8 (2016)
	–	OR (95% CI)	OR (95% CI)	OR (95% CI)	OR (95% CI)	OR (95% CI)
**Child exposure to SHS and parental active smoking**						
**No exposure to SHS at home from parents (a)**	Ref	1.17 (1.01–1.35)	1.65 (1.39–1.95)	2.85 (2.32–3.51)	2.24 (1.84–2.72)	3.66 (2.89–4.63)
**No maternal smoking during pregnancy (a)**	Ref	1.22 (1.06–1.40)	–	–	1.12 (0.97–1.31)	1.57 (1.33–1.86)
**None of parents is actively smoking (a)**	–	–	Ref	1.03 (0.94–1.12)	1.13 (1.03–1.24)	1.24 (1.13–1.36)
**Adverse health effects in children related to exposure to SHS**						
**Asthma never diagnosed by a doctor (b)**	Ref	–	–	0.76 (0.58–1.00)	0.74 (0.56–0.97)	1.64 (1.17–2.30)
**Neurodermatitis never diagnosed by a doctor (c)**	Ref	–	–	1.19 (1.05–1.35)	1.13 (0.99–1.28)	1.45 (1.26–1.66)
**Bronchitis never diagnosed by a doctor (c)**	Ref	–	–	1.01 (0.92–1.11)	1.23 (1.12–1.35)	1.36 (1.23–1.50)
**< 4 wheezing episodes in last 12 months (d)**	Ref	–	–	1.08 (0.79–1.47)	1.17 (0.85–1.61)	2.06 (1.40–3.04)

^*^SHS Second-hand smoking

(a) Adjusted for urbanization, parental education, mother and father employment, birth place of child and living in crowding conditions

(B) Adjusted for child gender, parental education level, mother and father employment status and living in crowding conditions

(c) Adjusted for urbanization, child gender, parental education level, mother and father employment status, child birth-place and living in crowding conditions

(d) Adjusted for child’s gender

Results from regression analysis of adverse health effects (Table 4), show odds of children never diagnosed by a doctor with asthma have increased by nearly two thirds in S8 compared to S1. Odds of children never diagnosed by a doctor with neurodermatitis are significantly higher in survey S6 and S8 compared to reference survey S1. Same applies for children never diagnosed by a doctor with bronchitis in S7 and S8 compared to reference survey S1. Children in S8 are twice as likely to have four or fewer wheezing episodes compared to reference survey S1. This indicates a decrease in prevalence of adverse health effects in children related to SHS, although the decreasing trend is not uniform over time.

## Discussion

The results demonstrate a decrease in exposure of Bavarian pre-school children to SHS during intrauterine development and early life years, as well as a decrease in active smoking in adults after implementation of the smoking ban in Bavaria in 2008. This decline was coupled with a decrease in reported adverse health effects in children related to their exposure to SHS.

In 2004, 40% of children worldwide were exposed to second-hand smoke with 1% of worldwide mortality attributable to this exposure, 28% of which are children [2]. These numbers suggest that substantial gains could be achieved if more policies aiming to protect children and non-smoking adults from exposure to SHS could be applied. Despite the intention behind enforcing a smoking ban in public places being to protect non-smokers, concerns were raised from policy makers about smoking displacement from public to private places where children and non-smoking adults live. Nevertheless, our results come in line with other studies refuting these concerns in different populations [16–19].

Although a clear decline in children’s exposure to SHS and parental active smoking could be noticed in our study, we cannot deliberately attribute this decline to the implementation of the smoking ban in Bavaria in 2008, but possibly to the improvement in education and increase in employment hours observed in our data over time. This improvement in socioeconomic conditions noticed in our data might not be a reflection to real social improvements, but keeping in mind the declining response rate over time from S1 to S8, this might introduce this socioeconomic improvement to our study population, with the assumption that socially privileged families are more aware about the negative health effects of smoking, and are keener to repeatedly take part in the survey with every new child entering his first school year, while socially disadvantaged groups would not be as keen to participate in the survey with every new child entering school.

While maternal smoking during pregnancy was linked in previous studies to diminished airway function in the new-born’s early life [37, 38], further exposure of the child to SHS increases the risk of respiratory infections and is responsible for two- to four-fold increased risk of wheezing illnesses and increased severity of asthmatic symptoms [38, 39]. In our study population, we noticed decreasing odds for maternal smoking during pregnancy, with numbers showing lower prevalence of health problems in children possibly linked to their decreasing exposure to SHS after 11 years from the smoking ban. This might be attributed to improved medical services [40] together with the decline in children’s exposure to SHS over time, also to the rising awareness from parents – especially the highly educated - towards the negative health effects of smoking and exposure to SHS.

Relative higher exposure of socially disadvantaged children to SHS has been described in several studies in Germany and internationally [3, 41, 42]. Studies performed on the effects of social disparities on exposure of children to SHS show that the relative decline in SHS exposure between different social groups over time is the same regardless of the social group [31, 43]. In consistence with other studies, we found a decline in children’s domestic SHS exposure in all educational groups, taking into account the decreasing proportion of parents with low education in our data across surveys, the decline seems to be relatively stronger in this group in which the de-normalising effect of the ban on the habit of smoking still seems to be effective, especially with the relatively higher odds of parents exposing their children to SHS in this group [24]. This decline promotes the implementation of the ban in communities where population educational level is not as high as in our investigated population, without fear of displacement of smoking to closed private places.

Single mothers are considered according to some studies as a socially disadvantaged group with higher risks of poverty and ill-health than other women [44]. Despite the increasing protective behaviour towards children’s exposure to SHS over time for both single and non-single mothers, in accordance with other studies [45, 46], after a descriptive analysis for our data, we observed that single mothers are more likely to expose their children to SHS at home than non-single mothers. As implementing the smoking ban does not show an increasing risk of exposing children of single mothers to domestic SHS, the smoking ban can be integrated with targeted campaigns raising awareness and improving the social environment of single mothers to reduce the relative high exposure of children of this social category to SHS [46].

Our study has some limitations that might introduce bias, despite the high response rate to the surveys included. The decreasing response rate over time coupled with change in social characteristics of respondents might indicate sampling bias under the assumption that higher educated parents are keener to participate in the survey. Also, parents of chronically ill children might have higher interest in filling questionnaires in order to report their children’s health problem, this could produce an overestimation of the reported diseases prevalence. Furthermore, relying on questionnaires to record children’s medical history can produce recall bias. It is also likely for social desirability bias to be introduced in questions asking parents if they expose their children to SHS hazards, leading to lower figures of actual percentage of parents who actually smoke and expose their children to SHS. To minimise information bias due to heterogeneity in questions quantifying the exposures across surveys, we always used the negative response as the event in all the investigated variables in our analysis (See supplementary, Methods section).

## Conclusions

Our results show that after 11 years from the smoking ban in all closed public amenities in Bavaria, there was no displacement of parental smoking to homes where children live. These findings come in line with studies investigating the effects of smoking bans in other countries. This would encourage investigating further implementation of smoking bans in private and public homes and vehicles in countries who have strict laws banning smoking in action, as well as promoting governments in other countries who have no strict smoking bans enforced to take similar steps. Our analysis results show that parental smoking habits are changing over time: parents are either not smoking at home in presence of children or are quitting smoking entirely. This change is noticed in all educational categories, taking into account the decreasing participation of the low education category into the surveys over time, that resulted in a profound decline in smoking in this group. Single mothers smoke more often in presence of children at home compared to non-single mothers, although the increase in not exposing children to SHS over time is noticed in both groups. Investigated health problems related to exposure of children to SHS (asthma, bronchitis, neurodermatitis and wheezing) are decreasing after 11 years from the smoking ban.

## Supplementary Information

**Additional file 1.**

## Data Availability

The databases used here in this study were exclusively available for the authors to access and use after an administrative permission. The datasets generated and/or analysed during the current study are not publicly available due confidentiality but are available from the corresponding author on reasonable request.

## Abbreviations

SHS: Second-hand Smoke.
OR: Odds Ratio
CI: Confidence Interval
GME: Health-monitoring Unit “Gesundheits-Monitoring-Einheiten”
