# Supplementary material for: Change in exposure of children to second-hand smoke with impact on children’s health and change in parental smoking habits after smoking ban in Bavaria – a multiple cross-sectional study
Source: BMC Public Health. 2021 Nov 20;21:2134. doi: 10.1186/s12889-021-12130-8 (PMC8605541; doi:10.1186/s12889-021-12130-8)
Supplement: Supplementary file 1 — Additional file 1. [file 12889_2021_12130_MOESM1_ESM.pdf]

Supplementary

Methods

In survey S4, 1070 questionnaires coming from Bamberg rural and urban areas were mixed by mistake, no assignment could be done for these questionnaires in survey S4 for "urbanisation" status as being rural or urban. This resulted in 20% missing data in the "urbanisation" status for this survey wave with the rest of the survey´s questionnaires (N = 4.266) having 39.1% coming from rural and 40.8% coming from urban areas in Bamberg. For inclusion of maximum number of questionnaires into the analysis, imputation of these missing data was done. Since the mixed questionnaires coming from rural and urban districts were randomly mixed by error, missing data pattern was proposed to be "missing completely at random", random assignment of the 1070 questionnaires to one of the values (rural/urban) was done using a random sequence generator. Results from regression analysis before and after imputation did not differ.

In our regression analysis, we always used the negative response – e.g. No, Never and no episodes of wheezing - as the event in our regression analysis and any other answer as reference in a binary variable. The purpose was avoiding any information bias emerging from heterogeneity in coding the variables from different possible answers on questions analysed across different surveys.

Figures

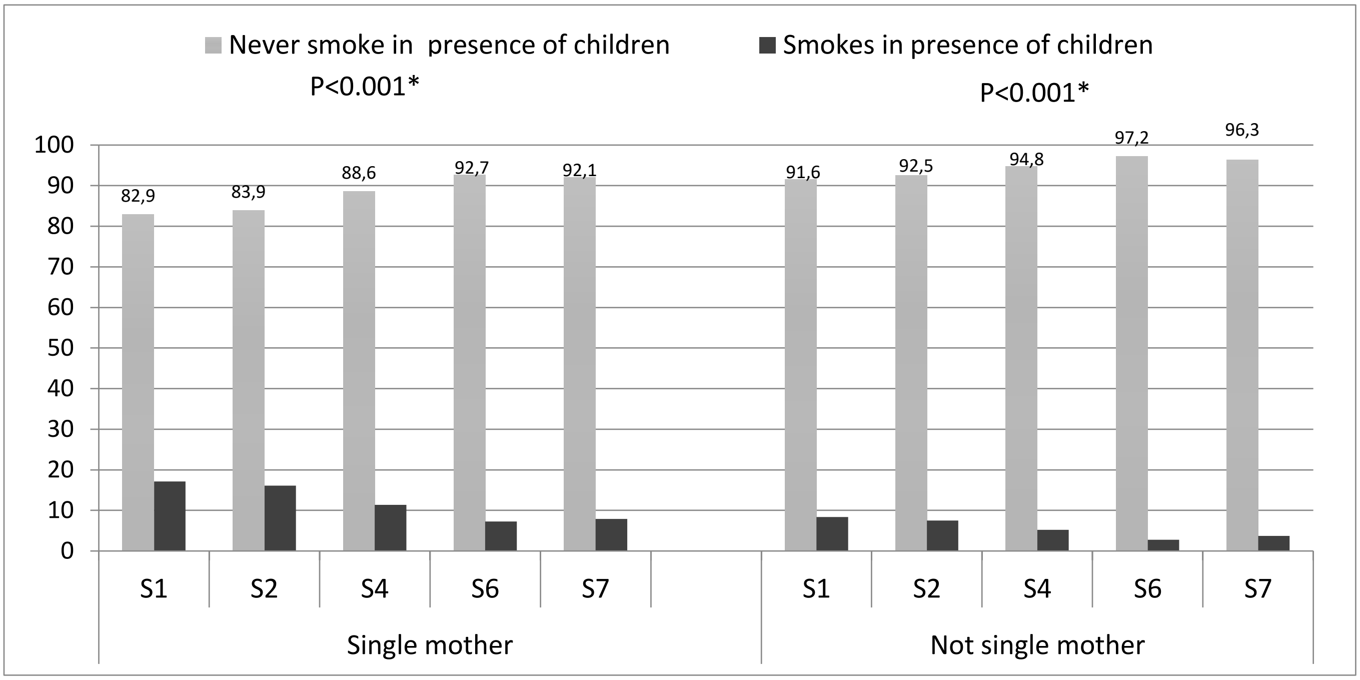

Figure 1 Exposure of children to domestic SHS stratified by being single mother.
*Design adjusted Rao-Scott chi-square test for independence.

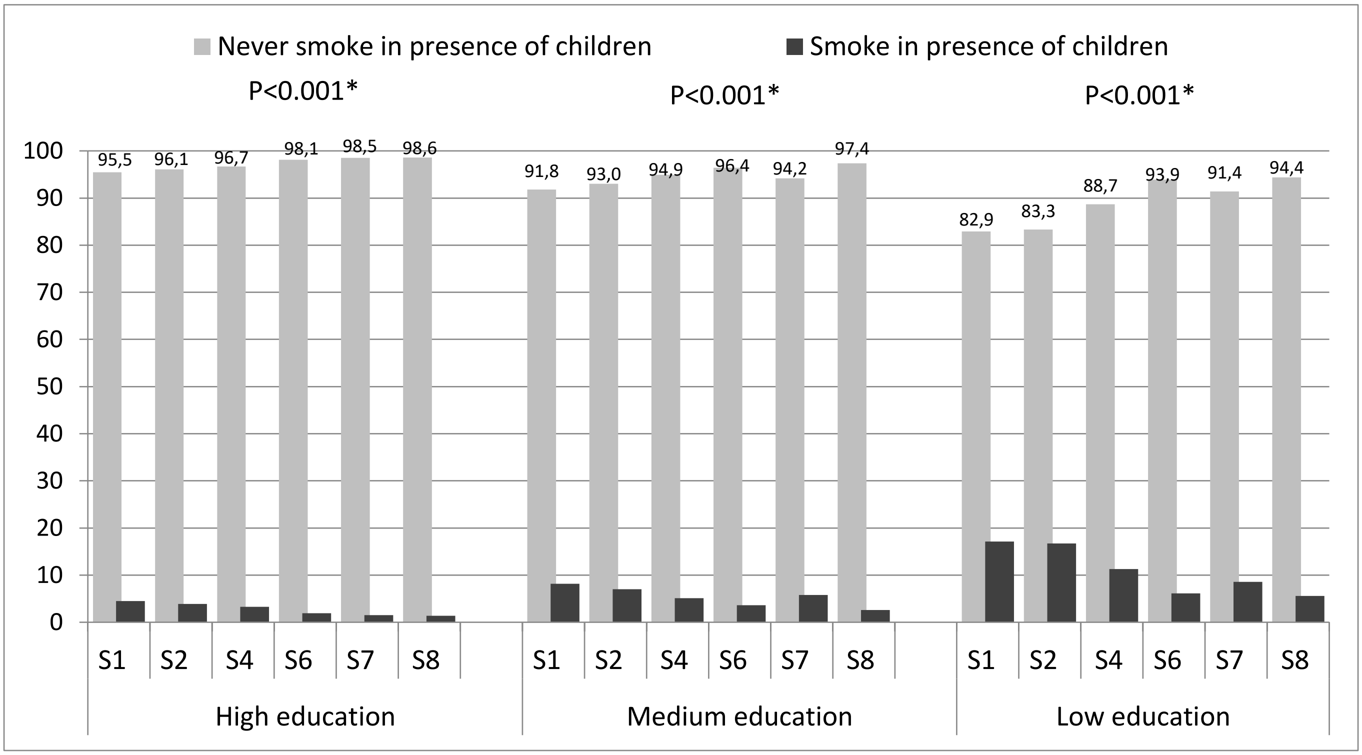

Figure 2 Exposure of children to domestic SHS stratified by parental education level.
*Design adjusted Rao-Scott chi-square test for independence.
